# Severe pulmonary hypertension associated with common arterial trunk in a 9- year- old child

**DOI:** 10.22088/cjim.11.4.454

**Published:** 2020

**Authors:** Saule Kabiyeva, SeyedFarzad Jalali, Farida Mindubayeva, Gaukhar Igimbayeva

**Affiliations:** 1 Karaganda Medical University, Karaganda, Kazakhstan; 2 Department of Cardiology, Babol University of Medical Sciences, Babol, Iran

**Keywords:** Congenital heart disease, Common arterial trunk, Pulmonary hypertension, Serum serotonin

## Abstract

**Background:**

Common arterial trunk (CAT) is a rare congenital heart disease, and often leads to the early development of pulmonary hypertension and disability. Among the critical structural heart defects, the frequency of CAT is 3%, which reflects the severe hemodynamic disturbances. The natural course of the disease is characterized by a high mortality rate up to 88% during the first year of life. We introduce a patient with common arterial trunk disease.

**Case presentation:**

The article describes a case of a 9-year old boy with a diagnosis of type I common arterial trunk (CAT) that rejected recommendations of many physicians for many years. At 7 months, the baby was diagnosed as CHD and at 3 years, a cardiac catheterization was performed and the final diagnosis was common arterial trunk, type I with defect of interventricular septum and pulmonary hypertension. However, at that time, parents refused the operation. The health status of the last examination was bad due to high pulmonary hypertension and chronic arterial hypoxemia. Drug therapy continued with bosentan, sildenafil, captopril, kudesang, spironolactone and aspirin.

**Conclusion:**

The late detection of children with CAT or late surgery leads to the rapid progression of pulmonary hypertension and significantly reduces the patient's chances for performing surgical correction of the defect.

Common arterial trunk (CAT) is a complex congenital heart disease (CHD), its frequency is 0.03-0.07 per 1000 live-born, and 1.1% - 4.6% of all CHD's. (1-3). Among the critical structural heart defects, the frequency of CAT is 3% (4, 5). The natural course of the disease is characterized by high mortality rate up to 88% in the first year of life (8) and the rapid development of pulmonary hypertension (9). CAT is a rare form of cyanotic congenital heart disease. It can be easily prenatal diagnosis. Depending on the anatomical variation, its clinical presentation is highly variable. The most common symptom and sign are mild cyanosis and significant tachypnea. Transthoracic echocardiography is used for diagnosis and detailed anatomical characterization. In complex pulmonary artery (PA) or aortic anomaly, other imaging methods such as cardiac catheterization, computed tomography angiography, or cardiac magnetic resonance imaging can be helpful. Complete repair is done for the patients in the neonatal period with resection of branch PAs from the CAT with placement of a right ventricular (RV)-to-PA conduit and patch closure of the ventricular septal defect. In general, surgical outcomes are excellent in most centers (10, 11). 

## Case Presentation

A child was from the 3rd pregnancy and 3rd delivery. Pregnancy was uneventful. Birth weight 3200g, and height was 53cm.There was a history of frequent respiratory viral infections and chicken pox. At 7 months, the child was diagnosed as CHD. After a survey in the local polyclinic, he was hospitalized at the National Scientific Medical Center of Astana city for complete examinations. Ultimately the diagnosis of common arterial trunk type I, was made and surgical correction was recommended; but because of high fever due to teething, the boy was discharged under the supervision of a cardiologist. At the age of 1, a cardiac surgeon consulted him and the correction of CHD was recommended again. However, the parents refused the operation, explaining their fear due to mortality following cardiac surgery. In January 2012, when he was 3 years of age, a cardiac catheterization was performed in the regional cardiac surgery center (RCSC) in Karaganda City and final diagnosis was common arterial trunk type I, with the defect of interventricular septum and pulmonary hypertension. The parents rejected cardiac surgery again. In Februry 2015, he was hospitalized in the National Scientific Cardiac Surgery Center for catheterization. Cardiac surgeons considered the surgical correction impossible due to severe pulmonary hypertension. 

The health status of the last examination was bad due to high pulmonary hypertension and chronic arterial hypoxemia. Consciousness was normal. Appetite was saved. Weight 20kg, height 115cm. moderate degree of acrocyanosis. Oxygen saturation 85%, heart rate and rhythm were normal, loud sound on the pulmonary area. systolic murmur along the left sternal border. Blood pressure 99/50 mm Hg. Electrocardiogram (ECG) showed 86/min normal sinus rhythm, right axis deviation with hypertrophy of the right ventricle. On the chest x-ray: The pulmonary fields were transparent. The roots are expanded and chopped off. The arterial bed enhanced the pulmonary picture. The sinuses were free. The diaphragm was in normal location. The shadow of the heart is extended to the left. C/T ratio was 61%.The vascular bed was dilated. Left ventricle was elongated and rounded.

Echocardiography showed common arterial trunk type I. Left-wing, right shaped heart. Atrium-ventricular was concordant. The aorta and the pulmonary artery were separated by a single trunk, with a single fibrous ring, in the center of the valve of common arterial trunk with moderate degree of regurgitation. Hypertrophy of the wall of the right ventricle 6 mm, mild tricuspid regurgitation. Systolic pressure in the right ventricle 70-75 mm Hg. Aorta dimension was 27-29 mm, IVS, 7 mm, PWLV, 6 mm, LV, 33 mm, LA, 39mm and LA area, 7.9 cm2, RV, 27 mm and area, 5.9 cm2. LVEDV, 31mm, LVESV, 11mm, LVSV, 20 ml, LVEF, 61%, RVSP, 75-80 mm Hg.

 Angiocardiography showed an arterial trunk, which is common for the right and left ventricles. CAT is located on the right, from its left edge extends to an expanded pulmonary artery. The branches of the pulmonary artery did not change, the peripheral drawing was enhanced. In the right phase, contrast is discharged from right to left at the level of an interventricular septal defect located right under the root of the common arterial trunk. In the left phase, discharge was not detected. The final diagnosis was common arterial trunk type I. Laboratory test showed serum serotonin levels were 327 ng/ml (normal:101-283), and platelet was 265 ng/ml. Drug therapy continued with bosentan, sildenafil, captopril, kudesang ,spironolactone , and aspirin.

## Discussion

Common arterial trunk belongs to rare congenital heart defects, and often leads to the disability of patients. The hemodynamic disturbances progress and in the first months, sometimes days, children die from progressive heart failure. Autopsy results indicate pulmonary insufficiency or stenosis of pulmonary arteries (12).

In hemodynamic study, the pressures in the aorta and the pulmonary artery become equal, and as a result of pulmonary overflow, pulmonary hypertension develops rapidly. It is generally accepted that the first days and months of life are ideal for surgical correction (13). In this case, the child’s parents rejected the operation due to its potential risk. Pulmonary hypertension significantly affects the rate of operation success and limits the possibility of surgical treatment (14). In our case, the child developed pulmonary hypertension, which was rapidly progressive.

Nowadays, simple and rapid laboratory tests are required to diagnose pulmonary arterial hypertension in CHD at initial stages timely surgical correction, this assay can prevent repeated cardiac catheterization (15). In recent decades, the role of the serotonin system in the pathogenesis of cardiovascular pathology has been widely discussed. There are works on measuring the level of serotonin synthesized by the cardiomyocytes of adult and newborn rats. Several scientists describe areas in the myocardium where serotonin accumulates, proving the role of serotonin in the development of heart of embryo and effect on the processes of differentiation and proliferation of organism (16). A number of authors believe that serotonin, by connecting to specific receptors on the endothelial and smooth muscle cells of vascular walls, regulates their tone, and affects myocardial metabolism (17).

There are publications that mention the concentration of plasma serotonin in children with CHD and pulmonary hypertension at the age of 4 increased by 2 times compared with children with CHD without pulmonary hypertension. Serotonin, which influences the regulation of vascular tone and myocardial metabolism, is involved in the development of pulmonary hypertension in CHD. Therefore, the determination of serotonin concentration in serum can be used as an early diagnostic marker in the presence of pulmonary hypertension in CHD (18). In conclusion the late detection of children with CAT leads to the rapid development of pulmonary hypertension and significantly reduces the patient's chances for surgical correction of defect.
